# Nomogram for predicting pathological response to neoadjuvant treatment in patients with locally advanced gastric cancer: Data from a phase III clinical trial

**DOI:** 10.1002/cam4.7122

**Published:** 2024-03-25

**Authors:** Han Shao, Nai Li, Yi‐hong Ling, Ji‐jin Wang, Yi Fang, Ming Jing, Zhi‐wei Zhou, Yu‐jing Zhang

**Affiliations:** ^1^ State Key Laboratory of Oncology in South China Sun Yat‐sen University Cancer Center Guangzhou People's Republic of China; ^2^ Department of Radiation Oncology Sun Yat‐sen University Cancer Center Guangzhou Guangdong People's Republic of China; ^3^ Department of Pathology Sun Yat‐sen University Cancer Center Guangzhou Guangdong People's Republic of China; ^4^ Department of Radiation Oncology, Shandong Cancer Hospital and Institute, Shandong First Medical University Shandong Academy of Medical Science Jinan People's Republic of China; ^5^ Department of Gastric Surgery Sun Yat‐sen University Cancer Center Guangzhou Guangdong People's Republic of China

**Keywords:** locally advanced gastric cancer, Neoadjuvant treatment, nomogram, pathological response

## Abstract

**Purpose:**

This study aimed to establish a nomogram using routinely available clinicopathological parameters to predict the pathological response in patients with locally advanced gastric cancer (LAGC) undergoing neoadjuvant treatment.

**Materials and Methods:**

We conducted this study based on the ongoing Neo‐CRAG trial, a prospective study focused on preoperative treatment in patients with LAGC. A total of 221 patients who underwent surgery following neoadjuvant chemotherapy (nCT) or neoadjuvant chemoradiotherapy (nCRT) at Sun Yat‐sen University Cancer Center between June 2013 and July 2022 were included in the analysis. We defined complete or near‐complete pathological regression and ypN0 as good response (GR), and determined the prognostic value of GR by Kaplan–Meier survival analysis. Eventually, a nomogram for predicting GR was developed based on statistically identified predictors through multivariate logistic regression analysis and internally validated by the bootstrap method.

**Results:**

GR was confirmed in 54 patients (54/221, 24.4%). Patients who achieved GR had a longer progression‐free survival and overall survival. Then, five independent factors, including pretreatment tumor differentiation, clinical T stage, monocyte count, CA724 level, and the use of nCRT, were identified. Based on these predictors, the nomogram was established with an area under the curve (AUC) of 0.777 (95% CI, 0.705–0.850) and a bias‐corrected AUC of 0.752.

**Conclusion:**

A good pathological response after neoadjuvant treatment was associated with an improved prognosis in LAGC patients. The nomogram we established exhibits a high predictive capability for GR, offering potential value in devising personalized and precise treatment strategies for LAGC patients.

## 1 INTRODUCTION

Gastric cancer ranks fifth among the most common malignant tumors worldwide and is the fourth leading cause of cancer‐related death.^1^ Traditionally, locally advanced gastric cancer (LAGC) patients were treated with radical surgical resection followed by adjuvant chemotherapy as the standard strategy. However, recent large clinical trials have increasingly recognized the benefits of neoadjuvant treatment for LAGC patients. The MAGIC study^2^ and the FNCLCC/FFCD^3^ study demonstrated that neoadjuvant chemotherapy (nCT) can lead to tumor downstaging, higher R0 resection rates, and improved survival compared to surgery alone. In the CROSS trial,^4^ neoadjuvant chemoradiotherapy (nCRT) significantly increased the R0 resection rate and prolonged survival. Furthermore, the RESOLVE study^5^ and the PRODIGY study^6^ confirmed that perioperative chemotherapy provided superior 3‐year disease‐free survival (DFS) for LAGC patients compared to adjuvant therapy.

However, the response to neoadjuvant treatment varies among patients, with only those showing a good response being associated with a better prognosis, while nonresponders do not gain survival benefits.^7^ Consequently, nonresponders may experience delays in optimal surgery timing, encounter treatment‐related adverse reactions, and incur additional medical costs. Tumor regression grade (TRG) is an effective method of assessing the outcome of neoadjuvant treatment based on pathological evaluation, with TRG0/1 indicating a more favorable prognosis.^7^, ^8^ However, despite obvious pathological regression, about 30% of patients still experience recurrence.^9^, ^10^ Recent studies have highlighted the importance of pathologic nodal status as an equally significant prognostic factor in LAGC patients undergoing neoadjuvant treatment.^11^, ^12^ The combination of TRG with ypN status may generate an evaluation method that is more comprehensive and reproducible to predict disease control.^13^, ^14^ However, there has not been much work in this area yet. For this study, we defined TRG0/1 (according to NCCN criteria) and ypN0 as good response (GR). Previous studies have primarily focused on establishing prediction models based on achieving pathological complete response (pCR) or TRG = 0, with enrolled patients receiving different chemotherapy regimens as preoperative treatment.^15^, ^16^ This prompted us to investigate whether GR has an independent influence on survival in patients who underwent preoperative treatments, including chemoradiation therapy.

Based on an ongoing prospective clinical trial, we conducted this study to identify pretreatment parameters that are capable of predicting GR to neoadjuvant treatment and establish a nomogram model. We hypothesized that a model to predict GR with a high probability (≥60%) could be developed, so the patients most likely to benefit could be identified and a more individualized, precise treatment strategy could be suggested.

## 2 MATERIALS AND METHODS

### 2.1 Study population and data collection

The current study was conducted based on the ongoing Neo‐CRAG study (NCT01815853), a multicenter, phase III randomized controlled trial of perioperative chemotherapy (nCT group) versus preoperative chemoradiotherapy (nCRT group) with postoperative chemotherapy for patients with resectable gastric cancer. All patients were newly diagnosed, untreated, potentially operable gastric cancer patients with stage III‐IVA (cT3N2–3M0, cT4aN+M0, and cT4bN any M0), a good performance status (Eastern Cooperative Oncology Group [ECOG] performance status score of 0–2), and no peritoneal implantation was confirmed by laparoscopy before nCT or nCRT. We conducted an analysis on patients treated at Sun Yat‐sen University Cancer Center between June 2013 and July 2022, who met the following criteria: (1) completion of preoperative treatment; and (2) undergoing gastrectomy. Patients who did not meet these inclusion criteria were excluded from the study. Ultimately, a total of 221 eligible patients were included in the analysis.

We collected all baseline clinicopathological information with potential predictive value, including gender, age, body mass index (BMI), biopsy pathological differentiation, presence of signet ring cell carcinoma component, tumor location, tumor staging information based on the American Joint Committee on Cancer (AJCC) 8th edition, as well as routine hematological and biochemical test results and tumor markers. For the tumor markers, we defined the upper limit of normal (ULN) as follows: CEA ≤5 ng/mL, CA199 ≤35 U/mL, and CA724 ≤6.9 U/mL. Additionally, we conducted follow‐up for each patient after gastrectomy.

### 2.2 Treatment

Patients in the nCT group received 3 cycles of neoadjuvant chemotherapy before surgery using the XELOX regimen: Oxaliplatin 130 mg/m^2^, intravenous drip, on day 1; and Capecitabine 1000 mg/m^2^, orally, on days 1–14; repeated every 21 days. Patients in the nCRT group also received three cycles of induction chemotherapy, with the latter two cycles combined with radiotherapy. Patients in the nCRT group also received three cycles of induction chemotherapy, with the latter two cycles combined with radiotherapy. Radical gastrectomy was performed 3–4 weeks and 6–8 weeks after the end of nCT and nCRT, respectively. Subsequently, patients continued to receive 3 cycles of adjuvant chemotherapy (XELOX regimen) 3–4 weeks after surgery.

### 2.3 Response assessment

In accordance with the same standards, all resection specimens were sent to the Department of Pathology for examination by the attending pathologist. The TRG criteria were selected according to the recent NCCN guideline (version 2, 2022).^17^ These criteria were adopted to reflect the recommendations of the College of American Pathologists (CAP), which designate a four‐category system for scoring treatment effect: grade 0 (complete response: no viable cancer cells), grade 1 (near‐complete response: single cells or rare small groups of cancer cells), grade 2 (partial response: residual cancer cells with evident tumor regression but more than single cells or rare small groups of cancer cells), and grade 3 (poor or no response: extensive residual cancer with no evident tumor regression).

We classified patients into two groups based on their TRG score and ypN status as follows: (1) GR group, consisting of patients with ypN0 status and complete or near‐complete tumor regression (Grades 0 or 1); (2) Poor response (PR) group, comprising patients with ypN0 status and partial or absence of tumor regression (Grade 2 or 3), as well as patients with ypN+ status.

### 2.4 Data analysis

The normality of the data was evaluated using the Kolmogorov–Smirnov test and normal probability plots. Non‐normally distributed parameters were presented as median (upper quartile to lower quartile) and analyzed using the non‐parametric Mann–Whitney rank test. Parameters that followed a normal distribution were expressed as mean ± standard deviation and analyzed using the *t*‐test. The Chi‐square test was used to analyze categorical variables. To compare progression‐free survival (PFS) and overall survival (OS) between the two groups, we utilized the Kaplan–Meier (K–M) method with log‐rank tests.

We conducted logistic regression analysis to identify predictive factors for GR, using odds ratio (OR) with 95% confidence interval (CI) to measure the associations. Preoperative parameters with a *p*‐value ≤0.05 in the univariate analysis were included in a multivariate logistic regression model. The final model was obtained using the Wald stepwise selection method, with an entry and removal probability set at *p* = 0.05. Subsequently, we constructed a nomogram based on the OR of the independent predictors. For validation, internal validation was performed using the bootstrap method. The calibration curve of the nomogram was plotted to illustrate the relationship between predicted and observed outcomes. The discriminative ability of the nomogram was assessed using a receiver operating characteristic (ROC) curve, and the area under the curve (AUC) was calculated to quantify its performance. Statistical significance was considered at *p* < 0.05. Data analyses were conducted using SPSS software version 26.0 (IBM, Armonk, NY, United States), GraphPad Prism 8.0 (GraphPad Software, San Diego, CA, USA), and R version 4.1.3 software (The R Foundation for Statistical Computing, Vienna, Austria).

## 3 RESULTS

### 3.1 Characteristics of the study population

The clinical characteristics of the study population were summarized in Table 1. The study cohort consisted of 164 males and 57 females. The median age at diagnosis was 60 years (range: 26–75). Over 80% of tumors were clinical T4 (178/221), and 97.7% of patients (216/221) had radiologically suspicious lymph node metastases. In total, 76% of the tumors were poorly differentiated adenocarcinomas (168/221), and 12.7% contained signet ring cell carcinoma (28/221). The median monocyte (MONO) count was 0.4 × 10^9^/L (range: 0.3 × 10^9^/L–0.5 × 10^9^/L). Abnormal elevation of pretreatment CA724 was observed in 29.4% of patients (65/221). During the study period, 110 patients (49.8%) underwent nCRT, and 111 patients (50.2%) underwent nCT.

**TABLE 1:** Patient characteristics and *p*‐value of univariate analysis.

Characteristics	Total (*n* = 221)	GR (*n* = 54)	PR (*n* = 167)	*p*‐value
Sex (*n* [%])				
Male	164 (74.2)	39 (72.2)	125 (74.9)	0.701
Female	57 (25.8)	15 (27.8)	42 (25.1)	
Age (years)	60 (50–65)	62 (53–66)	59 (50–65)	0.170
BMI (kg/m^2^)				
≦24.9	166 (75.1)	42 (77.8)	124 (74.3)	0.602
>24.9	55 (24.9)	12 (22.2)	43 (25.7)	
Location (*n* [%])				
Esophago‐gastric junction	43 (19.5)	12 (22.2)	31 (18.6)	0.678
Upper third	73 (33)	18 (33.3)	55 (32.9)	
Middle third	48 (21.7)	11 (20.4)	37 (22.2)	
Lower third	51 (23.1)	13 (24.1)	38 (22.8)	
Whole	6 (2.7)	0 (0)	6 (3.6)	
Lauren classification (*n* [%])				
Intestinal type	89 (40.3)	25 (46.3)	64 (38.3)	0.299
Non‐Intestinal type	155 (59.7)	33 (53.7)	122 (61.7)	
Differentiation (*n* [%])				
Well	53 (24)	19 (35.2)	34 (20.4)	0.027
Poor	168 (76)	35 (64.8)	133 (79.6)	
Signet ring cell (*n* [%])				
Yes	28 (12.7)	4 (7.4)	24 (14.4)	0.181
No	193 (87.3)	50 (92.6)	143 (85.6)	
Clinical T stage (*n* [%])				
T3	43 (19.5)	17 (31.5)	26 (15.6)	0.010
T4	178 (80.5)	37 (68.5)	141 (84.4)	
Clinical N stage (*n* [%])				
N0‐2	151 (68.3)	41 (75.9)	110 (65.9)	0.167
N3	70 (31.7)	13 (24.1)	57 (34.1)	
Blood type (*n* [%])				
Type O	84 (38)	24 (44.4)	60 (35.9)	0.629
Type A	70 (31.7)	17 (31.5)	53 (31.7)	
Type B	48 (21.7)	9 (16.7)	39 (23.4)	
Type AB	19 (8.6)	4 (7.4)	15 (9)	
WBC (10^9^/L)	6.30 (5.15–7.60)	6.3 (5.00–6.93)	6.30 (5.20–7.70)	0.470
NEU (10^9^/L)	4.00 (3.00–5.00)	4.00 (2.97–4.63)	3.90 (3.00–5.10)	0.565
HGB (g/L)	125 (105–140)	123 (108–140)	126 (103–140)	0.788
PLT (10^9^/L)	292 (226–351)	283 (222–346)	297 (226–354)	0.578
LYM (10^9^/L)	1.60 (1.30–2.00)	1.55 (1.30–2.00)	1.70 (1.30–2.00)	0.471
LYM%	0.26 (0.21–0.32)	0.26 (0.21–0.32)	0.26 (0.21–0.33)	0.747
MONO (10^9^/L)	0.40 (0.30–0.50)	0.40 (0.30–0.50)	0.40 (0.30–0.54)	0.010
ALB (g/L)	40.70 (37.65–42.85)	41.00 (39.20–43.85)	40.60 (37.50–42.60)	0.172
CRP (mg/L)	2.40 (0.90–7.25)	2.40 (0.80–6.48)	2.30 (0.90–7.60)	0.955
LDH (U/L)	149.70 (132.20–172.70)	155.20 (139.68–176.05)	148.10 (130.10–170.50)	0.076
NLR	2.47 (1.78–3.22)	2.34 (1.91–3.40)	2.50 (1.71–3.20)	0.963
LMR	4.00 (3.07–5.00)	4.08 (3.25–5.75)	3.91 (3.00–5.00)	0.099
PLR	176.67 (134.20–237.62)	176.49 (136.25–241.23)	176.67 (132.55–230.77)	0.824
CEA (ng/mL)				
≦5	166 (75.1)	44 (81.5)	122 (73.1)	0.213
>5	55 (24.9)	10 (18.5)	45 (26.9)	
CA199 (U/mL)				
≦35	178 (80.5)	43 (79.6)	135 (80.8)	0.854
>35	43 (19.5)	11 (20.4)	32 (19.2)	
CA724 (U/mL)				
≦6.9	156 (70.6)	44 (81.5)	112 (67.1)	0.043
>6.9	65 (29.4)	10 (18.5)	55 (32.9)	
Therapy (*n* [%])				
nCT	111 (50.2)	12 (22.2)	99 (59.3)	<0.001
nCRT	110 (49.8)	42 (77.8)	68 (40.7)	

Abbreviations: ALB, albumin; BMI, body mass index; CA199, carbohydrate antigen 199; CA724, carbohydrate antigen 724; CEA, carcinoembryonic antigen; CRP, C‐reactive protein; GR, good response; HGB, hemoglobin; LDH, lactate dehydrogenase; LMR, lymphocyte to monocyte ratio; LYM, lymphocyte; LYM%, lymphocyte%; MONO, monocyte; nCRT, neoadjuvant chemoradiotherapy; nCT, neoadjuvant chemotherapy; NEU, Neutrophil cell; NLR, neutrophil to lymphocyte ratio; PLR, platelet to lymphocyte ratio; PLT, platelets; PR, poor response; RBC, red blood cell; WBC, white blood cell.

### 3.2 Pathological analysis

Pathological examination of the surgical specimens revealed a pCR in 27 patients: 5 patients (4.5%) from the nCT group and 22 patients (20%) from the nCRT group (*p* < 0.001). Out of 221 patients, 75 (33.9%) achieved complete or near‐complete tumor regression (TRG0/1). Patients receiving nCRT had a significantly higher likelihood of achieving TRG0/1 compared to those receiving nCT (50% vs. 18%; *p* < 0.001). Nodal downstaging was achieved in 62.4% of the patients, with 75.5% in the nCRT group and 49.5% in the nCT group (*p* < 0.001). Additionally, ypN0 was observed in 93 patients (42.1%). Furthermore, 54 patients achieved a good response (GR, TRG0/1 plus ypN0): 42 (38.2%) in the nCRT group and 12 (10.8%) in the nCT group (*p* < 0.001). The PR group consisted of 167 patients: 21 patients achieved TRG0/1 plus ypN+, 39 achieved TRG2/3 plus ypN0, and 107 achieved TRG2/3 plus ypN+.

### 3.3 Survival outcomes

**FIGURE 1:**
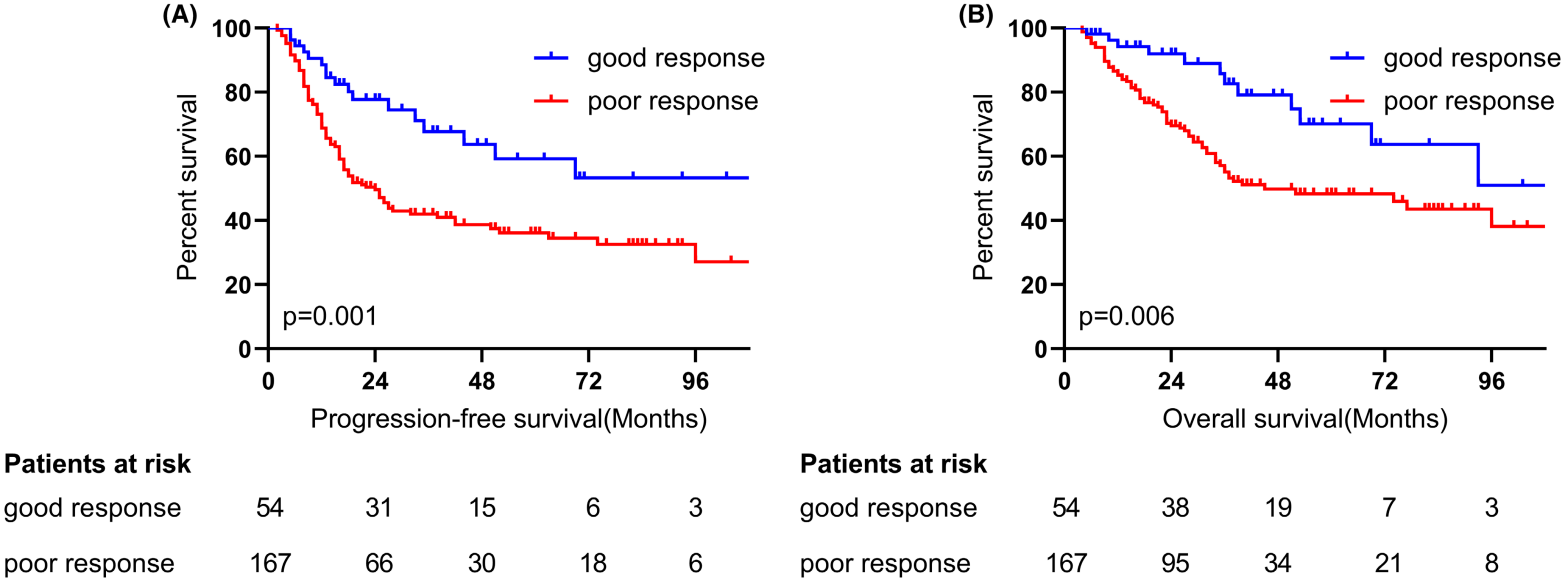
Survival according to pathological responses. Progression‐free survival (A) and overall survival (B) of patients between good response and poor response group.

To validate the prognostic value of TRG combined with ypN0, we conducted a survival analysis of different patient groups. The study population had a median follow‐up time of 42 months (range: 4–115 months). In the overall cohort, the median PFS was 32 months (95% CI, 19.8–44.2 months), and the median OS was 74 months (95% CI, 40.04–107.96 months). The K–M survival curve revealed that patients in the GR group demonstrated improved median PFS (GR vs. PR; not reached vs. 24 months; *p* = 0.001) and median OS (GR vs. PR; not reached vs. 45 months; *p* = 0.006) compared to patients with a poor response (Figure 1). The estimated 3‐year survival rates of patients with GR and PR were 82.6% and 55.2%, respectively. Consequently, we focused our attention on GR and established a prediction model.

### 3.4 Derivation of the prediction model

**FIGURE 2:**
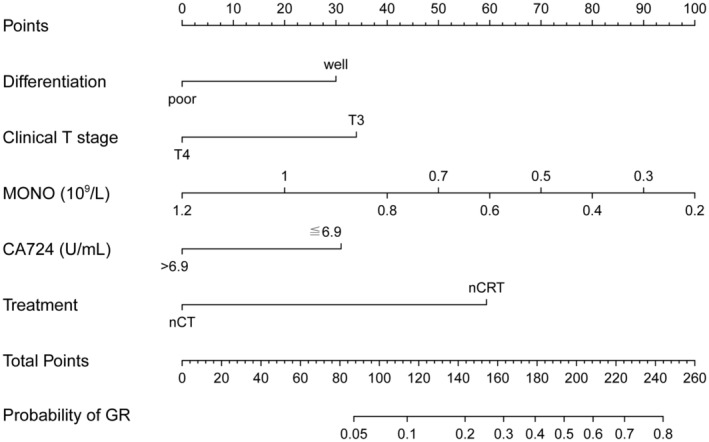
Nomogram for predicting GR to neoadjuvant treatment (GR, good response; nCT, neoadjuvant chemotherapy; nCRT, neoadjuvant chemoradiotherapy).

**FIGURE 3:**
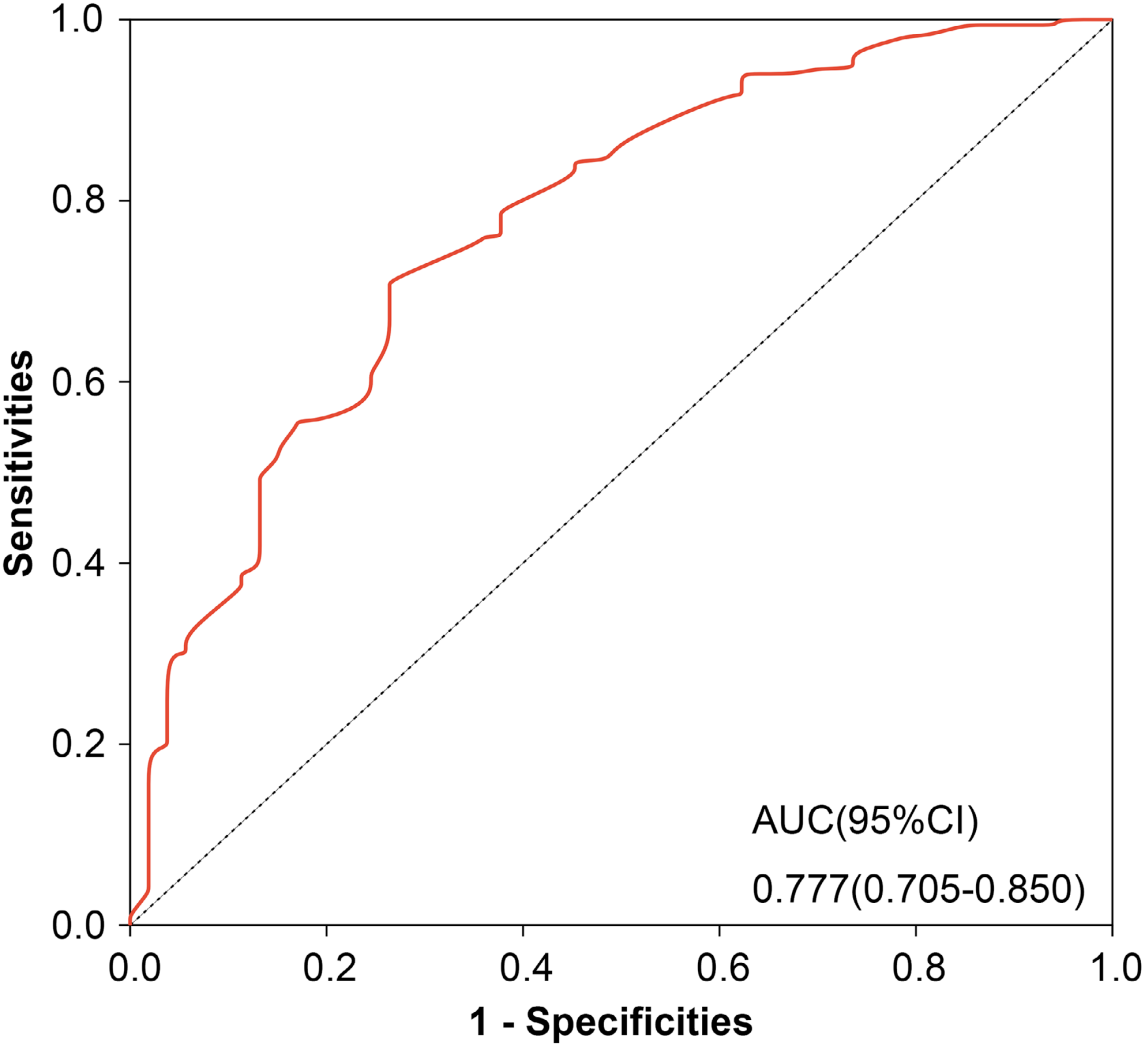
Receiver operating characteristic curve for the nomogram model (AUC, area under the curve; 95% CI, 95% confidence interval).

**FIGURE 4:**
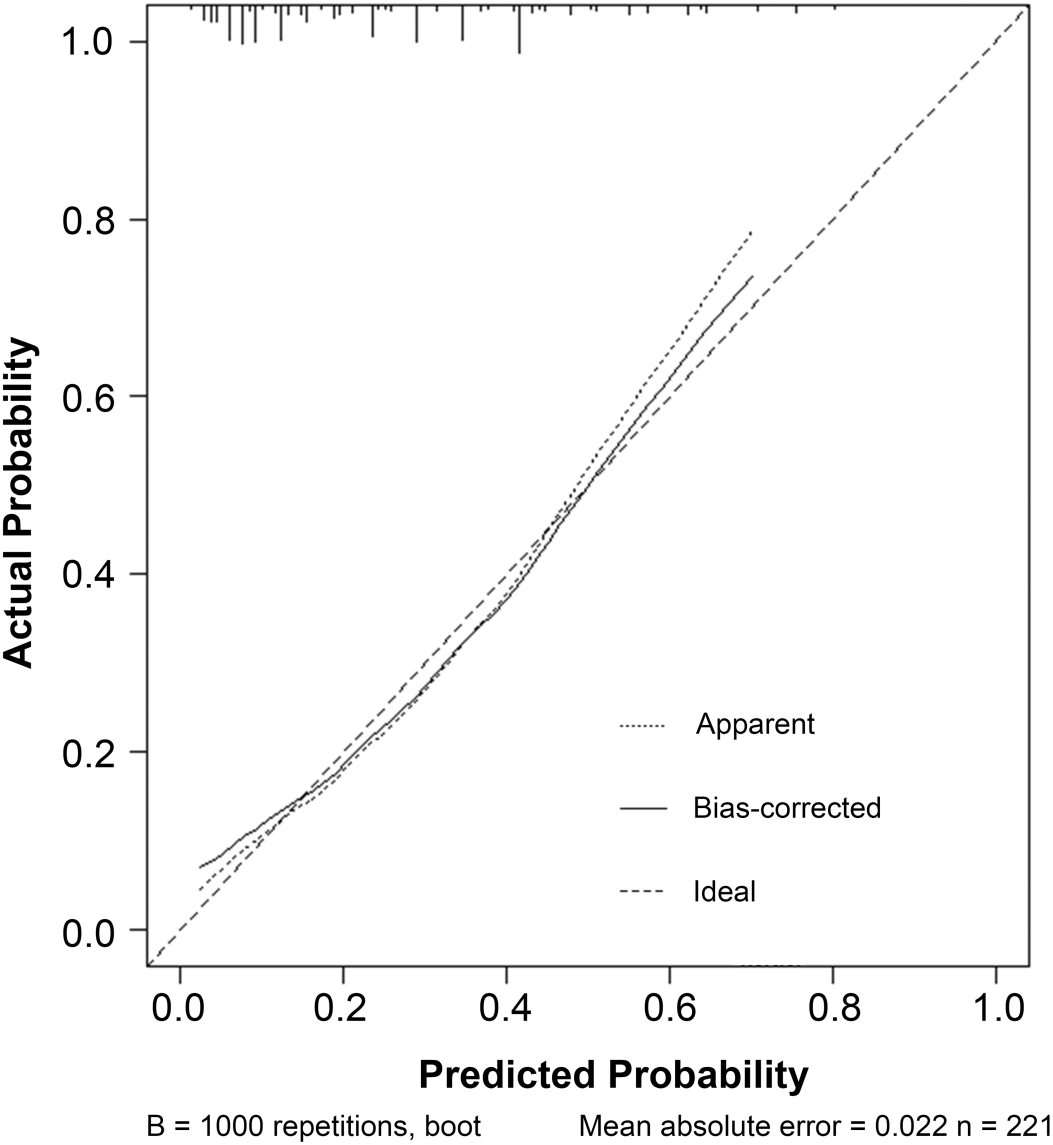
Calibration curve for the nomogram model.

Table 1 presents the univariate associations between clinical parameters and pathological response. Among the variables analyzed, five (tumor differentiation, clinical T stage, MONO count, CA724 level, and neoadjuvant modalities) showed significant differences (*p* < 0.05). Subsequently, using multivariate logistic regression analysis, we identified that GR was independently predicted by well tumor differentiation, a lower MONO count, clinical T3 (cT3), CA724 ≤6.9 U/mL, and the use of nCRT (Table 2). Based on these findings, we constructed a nomogram (Figure 2) that incorporates the five variables to enhance the probability of predicting GR up to 80% when a patient scores >240 points. The corresponding ROC curve (Figure 3) demonstrated an AUC of 0.777 (95% CI 0.705–0.850). After bias correction, the AUC remained high at 0.752, indicating excellent predictive discrimination. Additionally, internal validation was performed, and calibration curves were plotted to compare predicted and actual observations. As shown in Figure 4, the nomogram exhibited favorable statistical performance in predicting GR probabilities.

**TABLE 2:** Result of multivariate analysis.

Variables	*B*	*p‐*value	OR	95% CI
Differentiation (well/poor)	0.834	0.033	2.303	1.071–4.951
Clinical T stage (T3/T4)	0.933	0.023	2.543	1.141–5.670
MONO (10^9^/L)	−2.836	0.029	0.059	0.005–0.746
CA724 (≦/>6.9 U/mL)	−0.857	0.045	0.424	0.184–0.980
Therapy (nCRT/nCT)	1.640	0.000	5.157	2.443–10.888

Abbreviations: 95% CI, 95% confidence interval; *B*, beta coefficient; OR, odds ratio.

## 4 DISCUSSION

As treatment models for gastric cancer continue to evolve, neoadjuvant treatment has become increasingly important in comprehensive patient care. However, the challenge of tumor progression during the neoadjuvant treatment period remains. Identifying patients who are sensitive to neoadjuvant treatment is a crucial step in the decision‐making process for optimal treatment strategies.

Recently, various histologic, radiographic, and clinical measures of response to preoperative therapy have been analyzed in gastric cancer. Previous studies have reported associations between response to nCT and factors such as tumor location, differentiation, tumor‐infiltrating lymphocytes (TILs), and death‐associated protein‐3 (DAP‐3).^18^, ^19^ Additionally, radiomic information derived from computed tomography (CT) images has shown potential in identifying responsive patients to nCT.^20^ However, these findings were of limited clinical relevance due to the relatively complicated additional examination required or the small sample sizes involved. Chen et al.^15^ identified a correlation between higher lymphocyte ratios and carcinoembryonic antigen (CEA) levels, lower monocyte counts, and tumor differentiation grade with a higher pCR. Similarly, Xu et al.^16^ developed a prediction model for TRG = 0 using four predictors: CA199, CA724, tumor differentiation, and the short axis of the largest regional lymph node (LNmax). Although the indicators mentioned above were easily available, these studies were retrospective analyses of LAGC patients who received nCT with different chemotherapy regimens. In contrast, our study utilized data from a prospective phase III clinical trial, reducing potential biases in the study conclusions. Moreover, we defined and validated a novel and effective prognostic indicator for neoadjuvant treatment in LAGC. Ultimately, a nomogram based on five clinically routinely available predictors emerged, demonstrating a favorable discriminatory capability.

Both TRG and ypN status are equally important in predicting the prognosis of patients with LAGC. TRG, widely used in clinical practice, is determined by the percentage of remaining viable tumor cells, with a complete or near‐complete response indicating a better prognosis. However, conflicting results from some studies have shown that the status of lymph nodes after neoadjuvant treatment is the only independent prognostic factor for OS in gastric cancer patients.^11^, ^21^ It is important to consider that in some LAGC patients receiving neoadjuvant treatment, lymph nodes may remain positive despite good tumor regression at the primary site. This suggests possible variations in tumor biology between the stomach and metastatic lymph nodes, highlighting the limitation of evaluating treatment response based on a single factor. Martin‐Romano et al.^14^ demonstrated a significant survival benefit in patients with Becker grade 1a–b response combined with ypN0, compared to other groups. In our study, we defined “GR” as TRG0/1 and ypN0, and the survival analysis revealed that the GR group showed significantly improved OS and PFS compared to the PR group. Therefore, integrating the extent of tumor regression with ypN status may provide a more precise evaluation of patient response to neoadjuvant treatment and help identify individuals who could potentially benefit from it.

We identified five independent indicators that can predict GR in LAGC patients and developed a nomogram for prediction (with a concordance statistic of 0.752 after correction for optimism). The nomogram revealed that LAGC patients with well tumor differentiation, cT3, a low MONO count, and CA724 ≤6.9 U/mL before treatment were more likely to achieve GR. Prior research has suggested that well tumor differentiation is associated with a higher rate of pathological response to neoadjuvant treatment in gastric cancer.^15^, ^22^, ^23^ However, clinical T stage has consistently shown limited predictive value for histologic response in LAGC.^24^, ^25^, ^26^ In our study, we found that LAGC patients with cT3 were more likely to achieve GR compared to those with cT4. Importantly, most previous studies included a relatively low proportion of patients with cT4, whereas in our study, the proportion of cT4 patients exceeded 80%. Therefore, further research is needed to gain a deeper understanding of the relationship between the clinical T stage and the pathological response to neoadjuvant treatment in gastric cancer.

Monocytes are known to play a crucial role in tumor proliferation, angiogenesis, and progression, while also suppressing acquired immune responses and facilitating tumor invasion and migration. Consequently, patients with elevated monocyte counts often exhibit a poor prognosis.^15^, ^27^ In our study, we observed a correlation between elevated pretreatment monocyte levels and PR, which aligns with these previous findings.

Prior research has established serum CA724 levels as a valuable diagnostic and prognostic marker for gastric cancer, as well as an indicator for assessing surgical effect and predicting metastasis and recurrence.^28^ In the context of preoperative treatment, Sun et al.^29^ reported that a reduction in CA724 (>70%) can be indicative of a favorable pathological response to nCT. Additionally, Xu et al. demonstrated an association between CA724 ≤3.19 U/mL and pathological response (TRG = 0). In our study, we observed that CA724 ≤6.9 U/mL was linked to GR in gastric cancer patients after neoadjuvant treatment. In other words, lower CA724 levels are associated with an enhanced response to neoadjuvant treatment.

Locoregional recurrence is a critical determinant of prognosis in LAGC, and radiotherapy proves effective in controlling local lesions by precisely targeting and eliminating small clusters of cancer cells in the surrounding tissues. Radiotherapy also aids in eradicating cancer cells within the lymph nodes, increasing the chances of achieving a pCR. Consequently, the use of nCRT generally results in higher rates of complete primary tumor response and improved responsiveness of lymph nodes compared to chemotherapy alone.^14^, ^25^, ^30^ Consistent with previous data, our study demonstrated that patients in the nCRT group were more likely to achieve a GR.

However, we acknowledge certain limitations in our study, including the relatively small number of patients and the absence of external validation. Additionally, the inherent heterogeneity of gastric cancer may pose challenges in applying the predictive model. Various factors influence treatment response and outcomes, which warrant further exploration. In future research, incorporating more effective predictive indicators, such as molecular markers, will contribute to refining the predictive model and enhancing its accuracy. Despite these limitations, the use of convenient indicators has improved the practicality of our model. Moreover, it still offers a valuable reference for the development of future research models.

## 5 CONCLUSION

The present study developed a nomogram using five simple, readily available, and cost‐effective clinicopathological parameters to accurately predict GR in LAGC patients. These parameters include well tumor differentiation, low MONO count, cT3, CA724 ≤6.9 U/mL, and the acceptance of nCRT. The nomogram exhibited satisfactory discriminatory capability, as evidenced by a fairly high AUC in our cohort. This could potentially assist in devising personalized treatment strategies for LAGC patients. However, the applicability of the model still needs replication and external validation in multicenter, large‐scale studies.

## AUTHOR CONTRIBUTIONS

**Han Shao:** Conceptualization (equal); data curation (equal); formal analysis (equal); visualization (lead); writing – original draft (lead). **Nai Li:** Resources (equal); data curation (equal); software (equal). **Yi‐Hong Ling:** Data curation (equal). **Ji‐jin Wang:** Data curation (equal); formal analysis (equal). **Yi Fang:** Resources (equal); software (equal). **Ming Jing:** Resources (equal); software (equal). **Zhi‐wei Zhou:** Conceptualization (equal); writing – review and editing (equal). **Yu‐jing Zhang:** Conceptualization (equal); writing – review and editing (equal).

## FUNDING INFORMATION

This work was funded by grant 2012013 from the Sun Yat‐sen University Clinical Research 5010 Program.

## CONFLICT OF INTEREST STATEMENT

The authors declare no conflict of interest.

## ETHICS STATEMENT

The study was conducted in accordance with the Declaration of Helsinki, and approved by the by the Medical Ethics Committee of Affiliated Hospital of Sun Yat‐sen University Cancer Center (5010‐2012‐05‐03).

## INFORMED CONSENT STATEMENT

Informed consent to be included in the study was obtained from all patients.

## Data Availability

The data presented in this study are available from the corresponding author upon reasonable request.
